# Short-term transcutaneous electrical nerve stimulation reduces pain and improves the masticatory muscle activity in temporomandibular disorder patients: a randomized controlled trial

**DOI:** 10.1590/1678-77572016-0173

**Published:** 2017

**Authors:** Ana Paula de Lima FERREIRA, Dayse Regina Alves da COSTA, Ana Izabela Sobral de OLIVEIRA, Elyson Adam Nunes CARVALHO, Paulo César Rodrigues CONTI, Yuri Martins COSTA, Leonardo Rigoldi BONJARDIM

**Affiliations:** 1 Universidade Federal de Pernambuco, Departamento de Fisioterapia, Recife, PE, Brasil.; 2 Universidade Federal de Sergipe, São Cristóvão, SE, Brasil.; 3 Universidade Federal de Pernambuco, Programa de Pós-Graduação em Neuropsiquiatria e Ciências do Comportamento, Recife, PE, Brasil.; 4 Universidade Federal de Sergipe, Departamento de Engenharia Elétrica, São Cristóvão, SE, Brasil.; 5 Universidade de São Paulo, Faculdade de Odontologia de Bauru, Departamento de Prótese, Bauru, SP, Brasil.; 6 Universidade de São Paulo, Faculdade de Odontologia de Bauru, Departamento de Ciências Biológicas, Seção de Fisiologia da Cabeça e da Face, Bauru, SP, Brasil.

**Keywords:** Temporomandibular joint disorders, Transcutaneous electric nerve stimulation, Pain threshold, Electromyography

## Abstract

Studies to assess the effects of therapies on pain and masticatory muscle function are scarce.

**Objective:**

To investigate the short-term effect of transcutaneous electrical nerve stimulation (TENS) by examining pain intensity, pressure pain threshold (PPT) and electromyography (EMG) activity in patients with temporomandibular disorder (TMD).

**Material and Methods:**

Forty patients with myofascial TMD were enrolled in this randomized placebo-controlled trial and were divided into two groups: active (n=20) and placebo (n=20) TENS. Outcome variables assessed at baseline (T0), immediately after (T2) and 48 hours after treatment (T1) were: pain intensity with the aid of a visual analogue scale (VAS); PPT of masticatory and cervical structures; EMG activity during mandibular rest position (MR), maximal voluntary contraction (MVC) and habitual chewing (HC). Two-way ANOVA for repeated measures was applied to the data and the significance level was set at 5%.

**Results:**

There was a decrease in the VAS values at T1 and T2 when compared with T0 values in the active TENS group (p<0.050). The PPT between-group differences were significant at T1 assessment of the anterior temporalis and sternocleidomastoid (SCM) and T2 for the masseter and the SCM (p<0.050). A significant EMG activity reduction of the masseter and anterior temporalis was presented in the active TENS during MR at T1 assessment when compared with T0 (p<0.050). The EMG activity of the anterior temporalis was significantly higher in the active TENS during MVC at T1 and T2 when compared with placebo (p<0.050). The EMG activity of the masseter and anterior temporalis muscle was significantly higher in the active TENS during HC at T1 when compared with placebo (p<0.050).

**Conclusions:**

The short-term therapeutic effects of TENS are superior to those of the placebo, because of reported facial pain, deep pain sensitivity and masticatory muscle EMG activity improvement.

## Introduction

Transcutaneous Electrical Nerve Stimulation (TENS) has been used to control pain in patients with chronic temporomandibular disorders (TMD). However, the influence of this therapy on the tonus of masticatory muscles should be further investigated, considering that the evidence regarding the improvement of clinical parameters, e.g., reported pain, jaw movement and electromyography (EMG) activity, are divergent and controversial^4^ ^,^ ^6^ ^,^ ^18^ ^,^ ^22^.

It is well established that TENS can reduce patient-reported pain intensity in acute pain conditions^13^, but its efficacy is controversial in chronic pain^2^ ^,^ ^15^. On the other hand, to the best of our knowledge, there is no published study on the effects of TENS on the pressure pain threshold (PPT) of masticatory muscles. Nonetheless, findings from the cervical region have shown that TENS did not affect the PPT of upper trapezius trigger points^11^.

One study showed that the main effects of TENS on the muscular tonus of patients with TMD were: 1) reduction in muscle activity of the anterior portion of the anterior temporalis muscle, during resting posture of the jaw, and 2) increased muscle activity of the masseter muscles, during maximal voluntary contraction (clenching)^22^. Other study also confirmed TENS as effective in reducing the EMG activity of the anterior temporalis and masseter muscles during resting posture of the jaw^19^.

Mandibular kinematics may be subjected to changes that may compromise the muscle and articular functions in TMD patients^16^. Therefore, in addition to pain assessment, identifying dysfunctional EMG behaviors will also be useful in providing therapeutic management and preventing the progression of signs and symptoms^16^. Furthermore, pain reduction and improved function are commonly the proposed goals for treating chronic musculoskeletal pain^12^. Nevertheless, pain and muscle function are often evaluated separately, and studies to assess the effects of therapies on pain and masticatory muscle function are scant in the literature.

Based on the above, the aim of this study was to investigate the short-term effect of TENS by examining pain intensity, PPT and EMG parameters in subjects with myofascial TMD. Our initial hypothesis was that TENS is as effective in reducing facial pain as it is in improving masticatory muscle EMG activity of TMD patients.

## Material and Methods

### Participants and design

Academic staff and undergraduate students of both genders from the Federal University of Sergipe were eligible. They underwent clinical evaluation for examination of signs and symptoms of TMD and, after the inclusion and exclusion criteria assessment, participants were divided into two groups: (1) TENS placebo (n=20) and (2) TENS active (n=20). The study design was a randomized placebo-controlled trial. One investigator (R1) who did not participate in data collection performed the randomization with the aid of computed-generated combinatorial analysis, which was used to generate the random sequence. A second investigator (R2) performed the eligibility assessment and the group allocation was made by sealed and opaque envelopes (concealed allocation). Operational issues precluded masking of the researcher who applied the treatment. Then, the same investigator (R2) who performed the group allocation also performed the treatment. A third investigator (R3), blinded for group allocation, conducted the outcome assessments. Finally, a fourth investigator (R4) carried out the interpretation and analysis of results. It is important to note that the participants of this study were also blinded regarding the type of treatment.

Sample size of at least 20 subjects *per* group was determined based on pilot evaluations, which would be suitable to detect a pressure pain threshold (PPT) difference of 1.02 kgf/cm^2^, standard deviation (SD) of 1.12 kgf/cm^2^, considering the significance level of 5% (α), and sample power of 80% (β).

The Ethics Committee of the Federal University of Sergipe approved the study and the informed consent from each participant was obtained after full explanation of the research purposes and procedures.

### Inclusion and exclusion criteria

The inclusion criteria was the diagnostic of chronic painful TMD (at least six months of pain complaint) according to the Research Diagnostic Criteria for Temporomandibular Disorders (RDC/TMD), categories Ia (myofascial pain without limited mouth opening) or Ib (myofascial pain with limited mouth opening)^10^. The exclusion criteria were: a) a history of facial or head trauma, rheumatic and orthopedic pathologies, surgical procedures performed in the craniocervical region and neurological diseases; b) diagnostic of other chronic pain disorders, such as primary headaches, cervical pain disorders or fibromyalgia; use of oral contracptive; c) regular intake of medications, such as muscle relaxants, anticonvulsants, antidepressants and anxiolytics; d) any TMD treatment performed in the last three months; e) intake of any painkiller or oral contraceptive 24 h prior to the assessment. A detailed medical interview and clinical examination was carried out to evaluate inclusion and exclusion criteria. Also, the investigator responsible for the eligibility assessment (R2) was an orofacial pain specialist, trained and calibrated in the RDC/TMD examination technique.

### Outcomes

Reported facial pain intensity and PPT (primary outcomes), as well as EMG activity (secondary outcome), were assessed.

### Pain intensity

Visual analogue scale (VAS) was used to assess the intensity of current pain. VAS measures the painful experience using a straight line of 100 mm, with the left margin anchored by the term “no pain”, and the right, by the term “worst imaginable pain”^3^.

### Pressure Pain Threshold (PPT)

PPT was conducted using an algometer (Kratos^®^, Cotia, SP, Brazil) containing a rod with a circular flat surface of 1 cm^2^ coated with soft rubber. The patient was positioned comfortably in a sitting position, with muscles relaxed. The evaluator then placed the end of the circular surface of the algometer perpendicular to the skin and applied a steadily increasing pressure of 0.5 kg/cm^2^/second. The patient was instructed to verbalize the moment when the pressure exerted caused a painful sensation. The following sites were assessed: masseter muscles, anterior temporalis muscle, and sternocleidomastoid (SCM) and upper trapezius (bilaterally) muscles, in addition to the lateral pole of the jaw. The average of two trials was considered the PPT. There was a two-minute interval between the first and second measurements at the same muscle site, and a five-second interval between the measurements of one muscle site and the other^7^.

### Electromyography (EMG)

The surface EMG record was obtained using a Miotec^®^, model Miotool 400 4-channel system, which acquires the EMG signals (14-bit), with electrical isolation of 3,000 volts, high EMG signal representation across all channels (2000 samples/second per channel), rejection of 110 dB common mode and low noise level <2 LSB (Low Significant Bit). The acquisition of EMG signals was performed using Miograph software with a 2000 Hz sampling frequency, 20-500 Hz bandpass filter with interference eliminated by the Notch Filter^25^. In performing the data analysis, the authors considered the amplitude of the electrical potential in microvolts (uV), expressed by the root mean square (RMS)^1^. Disposable and circular electrodes by Meditrace^®^ were used, with a 20 mm distance between the poles. The ground electrode was placed in the lateral epicondyle of the left elbow of all volunteers.

A muscle function test performed before placing the electrodes served to identify the center of the muscles to be analyzed. The electrodes were placed parallel to the muscle fiber of these muscles, equidistant from the muscle origin and insertion. Before the exam itself, subjects were asked to perform a maximum voluntary contraction (MVC), supported by a five-second isometric contraction of the masseter muscles and anterior temporalis muscle, in order to conduct normalization of the data, interpreted subsequently with the MATLAB.

EMG signal was captured in three tasks: in the mandibular rest position (MR), during MVC and during habitual chewing (HC). In measuring HC, Trident^®^ gum was used for 20 chewing cycles^1^.

### Assessments

All evaluations were made in three assessment times: baseline (T0), immediately after (T1) and 48 hours after (T2) treatment. The participants were assessed according to the group allocation (intention-to-treat analysis).

### Treatment (Active TENS)

The volunteer was positioned in dorsal decubitus with knees supported on a triangular pillow placed between the volunteer’s chest and head. Electrodes were placed on both the masseter muscles and the anterior temporalis muscle beams, considering the same references that were used for electrode placement of the electromyography surface exam.

Before muscle stimulation, all participants were informed of the different types of interventions being tested in the research and of the perceived sensation of paresthesia from the electrodes, ranging from unnoticeable to hardly noticeable or very noticeable. The TENS device used was model Neurodyn Sapphire Compact Line, by Ibramed^®^, with two previously calibrated channels, and circular adhesive electrodes by Valutrode^®^, 3 cm in diameter. The parameters used in this study were: pulse duration only up to sensory activation (<100 us) and high intensities, but with an established limit to prevent muscle contraction and allow maximum comfort during the 50 minutes of therapy.

The total time of treatment was 50 min using variations of low and high frequency (VHF), with a sweep of 4 Hz (first 25 min) and 100 Hz (last 25 min). This application protocol was based on previous evidence, which report different and complementary analgesic mechanism when adopting high and low frequencies^9^ ^,^ ^20^ ^,^ ^24^ ^,^ ^26^. The TENS device was connected to a placebo device with a selector key that was switched off, without the volunteers knowing this, in such a way not to allow them to distinguish between test procedure and placebo.

### Treatment (placebo TENS)

Placebo device was developed in the Electrical Engineering Laboratory of the Federal University of Sergipe (GPRUFS), Robotics Research Group. The placebo equipment allowed the passage of current to the participant for only a short period of time (40 seconds). The current was gradually reduced in such a way that the receiver would not be able to perceive the interruption in the stimulus. The placebo device had an internal resistance with values close to human body resistance. This system did not allow the electrical stimulation device to recognize that the current was not going through the individual, thus avoiding a false recording of non-contact electrodes.

Placebo procedure was performed with the placebo group positioned in the same way as the test group (active TENS), and with the same current parameters used for the test group. All the patients were told was that they were participating in a study involving the possibility of a placebo treatment.

### Statistical analysis

Quantitative variables, i.e., age, body mass index – body mass divided by the square of the body height – (BMI), VAS, PPT and EMG at different tasks were presented as mean and standard deviation (SD). Variables were tested for data normality by the Kolmogorov-Smirnov test, and presented normal distribution (p>0.050).

The effect of TENS on VAS, PPT and EMG activity at different tasks (MR, MVC and HC) within and between the groups over time (before, immediately after and 48 hours after application of TENS) was computed using two-way ANOVA for repeated measures followed by Tukey’s *post*-test. The significance level was set at 5%. In addition, the effect size of significant comparisons was computed according to Cohen’s kappa coefficient (d). Cut-off points can indicate small (d=0.20), moderate (d=0.50) and large (d=0.80) effects^5^. Missing data in consequence of dropouts were excluded from the final analysis.

## Results

**Figure 1:**
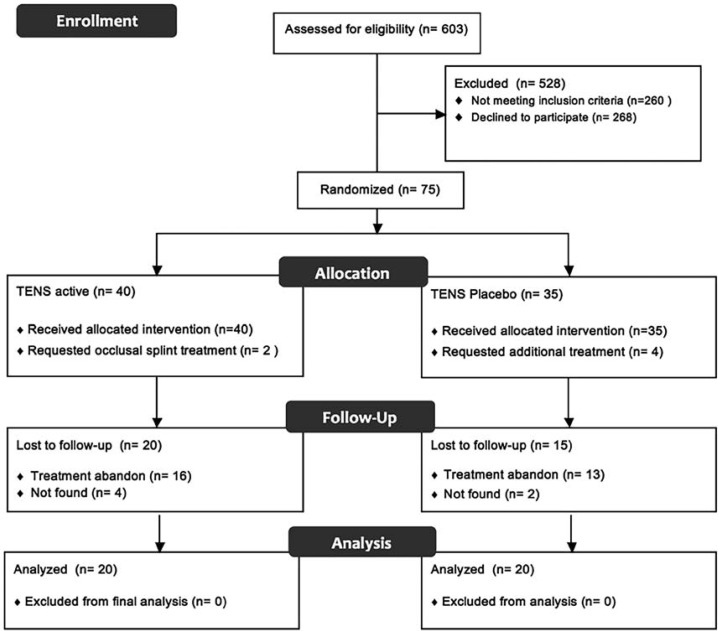
Flow diagram of the study stages from recruitment to final analysis

**Figure 2:**
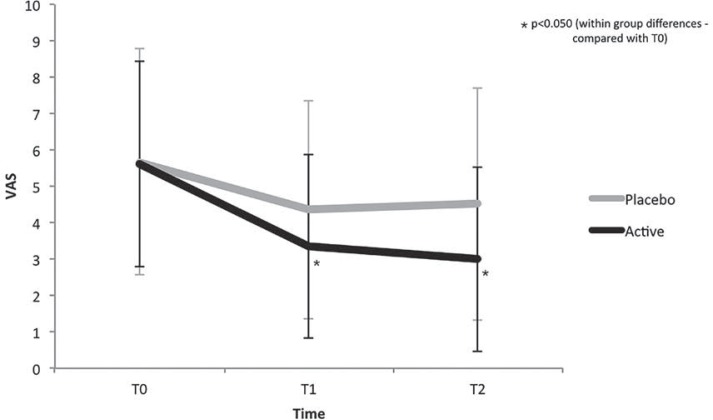
Mean of the pain intensity at all assessment times

The flow of participants is described in Figure 1. In addition, Table 1 describes the general characteristics of the participants who were included in the final analysis. No significant difference was observed between the groups for gender, age and BMI (p<0.050). Moreover, no between-group difference was found in the VAS values at any assessment time (p>0.050). However, there was a significant decrease in the VAS values at T1 (d=-0.79) and T2 (d=-0.92) when compared with T0 values (within-group differences) only in the active TENS group (Figure 2).

**Table 1:** Baseline characteristics of the sample

	Placebo TENS (n=20)	Active TENS (n=20)	p-value
Gender - n(%)			ns
Female	15 (75%)	15 (75%)	
Male	5 (25%)	5 (25%)	
Age (years) - Mean (SD)	24.15 (3.01)	25.10 (3.87)	ns
BMI (Kg/m^2^) - Mean (SD	23.29 (2.28)	24.45 (5.80)	ns
Axis I RDC/TMD - n(%)			
IA	12(60%)	11(55%)	
IA/IIA	7(35%)	8(40%)	
IB	1 (5%)	0(0%)	
IB/IIB	0 (0%)	1(5%)	

ns=non-significant (p>0.050)

RDC/TMD=Research Diagnostic Criteria for temporomandibular disorders.

IA=myofascial pain; IIA=disk displacement with reduction; IB= myofascial pain without

mouth opening limitation ;IIB=myofascial pain with mouth opening limitation

BMI= body mass index

VAS=visual analogue scale, T0=baseline, T1=immediately after treatment and T2=48 hours after treatment. Error-bars indicate the standard deviation of the mean

PPT values of the anterior temporalis (d=1.13), TMJ (d=1.23) and SCM (d=1.69), were significantly higher in the active TENS and at T1 when compared with placebo, but also for the masseter (d=1.05) and SCM (d=1.38) at T2 (p<0.050) (Table 2). There was no significant increase in the PPT values in any of the assessment times considering the placebo group (p>0.050). However, there was a significant increase in the PPT values of masseter (d=0.57), anterior temporalis (d = 0.68), TMJ (d=1.10) and SCM (d=1.46) at T1 when compared with T0, and of masseter (d=0.46) at T2 compared with T1 in the active TENS group (p<0.050) (Table 2).

**Table 2:** Mean (SD) of the pressure pain threshold (PPT) values (Kgf/cm2) of masticatory muscles, temporomandibular joint (TMJ) and cervical muscles

	Baseline (T0)	Immediate (T1)	48 hours (T2)
Placebo TENS (n=20)			
Masseter	1.69 (0.74)^aA^	1.62 (0.62)^aA^	1.71 (0.67)^aA^
Anterior Temporalis	1.83 (0.80)^aA^	2.02 (0.88)^aA^	2.09 (0.92)^aA^
TMJ	1.84 (0.31)^aA^	2.01 (0.47)^aA^	1.96 (0.41)^aA^
Sternocleidomastoid	1.32 (0.45)^aA^	1.46 (0.58)^aA^	1.49 (0.64)^aA^
Upper Trapezius	2.61 (0.85)^aA^	2.77 (0.94)^aA^	2.69 (1.09)^aA^
Active TENS (n=20)			
Masseter	1.76 (0.59)^aA^	2.10 (0.59)^bA^	2.37 (0.58)^cB^
Anterior Temporalis	2.01 (0.69)^aA^	2.79 (0.38)^bB^	2.47 (0.41)^cA^
TMJ	1.97 (0.63)^aA^	2.56 (0.42)^bB^	2.30 (0.62)^bA^
Sternocleidomastoid	1.57 (0.57)^aA^	2.23 (0.28)^bB^	2.18 (0.30)^bB^
Upper Trapezius	2.93 (1.21)^aA^	3.43 (0.95)^bA^	2.70 (0.75)^bA^

Different small letters in the same row represent significant within-group differences (p<0.050)

Different capital letters in the same column represent between-group differences (p<0.050)

EMG activity of the masseter (T0, d=-2.57, T1, d=-4.58 and T2, d=-6.26) and anterior temporalis (T0, d =-2.03, T1, d=-3.69 and T2, d=-3.12) were significantly lower in the active TENS during MR at all assessment times when compared with placebo (p<0.050) (Table 3). Nevertheless, a significant EMG activity reduction of the masseter (d=-4.98) and anterior temporalis (d=-3.77) was presented in the active TENS during MR at T1 assessment when compared with T0 (p<0.050), whereas the placebo increased MR EMG activity of the masseter (T0, d=0.37 and T1, d=0.22) at T2 assessment when compared with T0 and T1 (p<0.050) (Table 3).

**Table 3:** Mean (SD) of the electromyography values in microvolts expressed as Root Mean Square for the masseter and the anterior temporalis at different tasks

	Baseline (T0)	Immediate (T1)	48 hours (T2)
Placebo TENS (n=20)			
Mandibular rest			
Masseter	7.70 (1.52)^aA^	8.02 (1.44)^aA^	8.42 (1.11)^bA^
Anterior Temporalis	8.51 (1.59)^aA^	8.82 (2.02)^aA^	8.65 (2.29)^aA^
Maximum Voluntary Contraction			
Masseter	160.66 (49.97)^aA^	217.20 (50.70)^bA^	190.65 (47.77)^aA^
Anterior Temporalis	152.19 (52.70)^aA^	80.42 (26.60)^bA^	116.30 (39.64)^bA^
Habitual Chewing			
Masseter	19.94 (3.33)^aA^	23.14 (3.56)^aA^	23.99 (3.35)^aA^
Anterior Temporalis	19.45 (2.58)^aA^	24.08 (6.00)^aA^	23.22 (5.28)^aA^
Active TENS (n=20)			
Mandibular rest			
Masseter	4.84 (0.40)^aB^	2.92 (0.37)^bB^	3.22 (0.38)^bB^
Anterior Temporalis	5.78 (1.04)^aB^	2.89 (0.30)^bB^	3.53 (0.37)^bB^
Maximum Voluntary Contraction			
Masseter	134.64 (21.96)^aA^	205.82 (43.84)^bA^	179.13 (52.77)^bA^
Anterior Temporalis	140.32 (19.44)^aA^	203.23 (59.49)^bB^	164.27 (45.83)^aB^
Habitual Chewing			
Masseter	22.86 (2.41)^aA^	45.14 (9.82)^bB^	28.35 (7.78)^cA^
Anterior Temporalis	20.36 (3.05)^aA^	44.10 (9.63)^bB^	27.16 (6.50)^cA^

Different small letters in the same row represent significant within-group differences (p<0.050)

Different capital letters in the same column represent between-group differences (p<0.050)

EMG activity of the anterior temporalis (T1, d=2.66 and T2, d=1.11) was significantly higher in the active TENS during MVC at T1 and T2 when compared with placebo (p<0.050) (Table 3). In addition, a significant increase in the EMG activity of the masseter (TENS active, d=2.05 and TENS placebo, d=1.12) and anterior temporalis (TENS active, d=1.42) was also observed in the active TENS and placebo during MVC at T1 when compared with T0 (p<0.050), whereas a significant reduction in the EMG activity of the anterior temporalis (d=-1.71) in the placebo was observed during MVC at T1 when compared with (p<0.050) T0 (Table 3).

EMG activity of the masseter (d=2.97) and anterior temporalis (d=2.49) muscle was significantly higher in the active TENS during HC at T1 when compared with placebo (p<0.050) (Table 3). Moreover, a significant increase in the EMG activity of the masseter (T1, d=3.11 and T2, d=0.95) and anterior temporalis (T1, d=3.32 and T2, d=1.33) was observed only in the active TENS during HC at T1 and T2 when compared with T0 (p<0.050) (Table 3).

## Discussion

This study demonstrated that mostly of TENS hypoalgesic properties and improvement in EMG activity in subjects with myofascial TMD are not placebo effects. The main findings were: (a) short-term (T1 and T2) reduction in pain intensity; b) short-term increase in PPT values; (b) immediate (T1) reduction in MR EMG activity and short-term increase in MVC and HC EMG activity.

The evidence is controversial regarding the TENS effects of pain reduction in chronic pain disorders, though electrical nerve stimulation modalities in general are considered an effective treatment for chronic musculoskeletal pain. In particular, TENS seems to be effective on reducing pain in TMD patients. However, positive outcomes are generally not reported immediately after the application, but rather they are proposed as cumulative effects. Our study presented both, immediate (T1) and cumulative effects of TENS application (T2), which could be partially explained by the use of high frequency TENS. Previous evidence has reported immediate effects on TMD pain when applying only high frequency TENS. However, as far as we know, there is no published study comparing high and low frequency TENS in TMD patients, which warrants further researches.

A proper TMD evaluation would include muscle tenderness investigation, which can be done by manual palpation or with the aid of more standardized and reliable techniques, e.g., PPT assessment^21^. Previous evidence has shown inconsistent results of TENS effect on muscle tenderness in TMD patients, with reports of no effects on masseter and anterior temporalis pain upon palpation after four weeks^14^, but also with positive effects on pericranial muscle tenderness score (PTS) after 10 weeks^8^. Interestingly, this is the first study to report that TENS can increase PPT of masticatory muscles, which reinforce the claimed positive effects of electrical therapy on muscle pain.

The pain adaptation model^17^ advocates that chronic muscle pain can reduce agonist muscle contraction and an increase antagonist muscle activity, in order to protect the agonist from new injuries^19^ ^,^ ^23^. Our findings for EMG activity reduction at MR and increase at MVC and HC may point out TENS as an important contribution to lowering energy expenditure in maintaining jaw rest, and to improving the power efficiency of jaw functions in patients with TMD. However, previous reports showed that high frequency TENS does not influence EMG activity of masseter and anterior temporalis muscles at clenching in TMD patients^22^. Discrepancy in the TENS application protocol could explain such differences, considering that variations in the stimulation frequency of TENS could be considered important to obtain unlikeness at MR or MVC^22^. In addition, since this is the first study to demonstrate TENS efficacy on muscle pain with the aid of evaluation of muscle EMG activity at three different tasks, more researches are required to support our findings, mainly in the long-term assessment.

Such positive short-term effects of TENS on muscle pain and function could be related with the alternate frequencies protocol adopted in our study. This application protocol was based on previous evidence, which report different and complementary analgesic mechanism when adopting high and low frequencies^9^ ^,^ ^26^. High frequency TENS has been associated with segmental pain inhibition at neurons located in the dorsal horn and it can reduce nociceptive substances released in peripheral tissues^24^. In addition, low frequency TENS has been associated with release of enkephalins and β endorphins within the descending pain modulation system^24^. Nevertheless, it is important to note that this is the first report in the literature on the use of alternate frequencies in TMD patients. Considering that there is no sound conclusions regarding the optimal TENS protocol, further investigation is required not only to determine superior efficacy of alternate frequency TENS therapy for myofascial TMD but rather to establish guidelines for TENS application.

The strengths of this study are mainly related with the systematic assessment of myofascial TMD pain and muscle function using valid and reliable methods. On the other hand, some limitations that can be highlighted in this study were: a) lack of a long-term assessment; b) lack of a control group without any treatment, since aspects related to fluctuation periods and pain remission in TMD patients must be considered before any final judgment is made regarding therapeutic efficacy, although ethical implications of such procedure should be considered; c) lack of a control group without TMD, which could also elucidate the effects of TENS on asymptomatic muscles; d) risk of treatment bias, because the researcher who applied the treatment was aware about the group allocations.

## Conclusions

Short-term therapeutic effects of TENS are superior to those of the placebo, because of the reported facial pain, deep pain sensitivity and masticatory muscle EMG activity improvement. Accordingly, we recommend the use of TENS as an effective option for short-term management of masticatory myofascial pain. However, further investigations are required to determine if this efficacy is also present in the long-term effects.
